# Gemcitabine and docetaxel as first-line treatment for advanced urothelial carcinoma: a phase II study

**DOI:** 10.1038/sj.bjc.6602378

**Published:** 2005-02-01

**Authors:** A Ardavanis, D Tryfonopoulos, A Alexopoulos, C Kandylis, G Lainakis, G Rigatos

**Affiliations:** 1 1st Department of Medical Oncology, St Savas Anticancer Hospital, 171, Alexandras Avenue, 11522 Athens, Greece

**Keywords:** gemcitabine, docetaxel, bladder cancer, transitional cell carcinoma

## Abstract

The purpose of the study was to investigate the toxicity and efficacy of the combination of gemcitabine and docetaxel in untreated advanced urothelial carcinoma. Patients with previously untreated, locally advanced/recurrent or metastatic urothelial carcinoma stage-IV disease were eligible. Patients with Performance status: PS ECOG >3 or age >75 years or creatinine clearance <50 ml min^−1^ were excluded. Study treatment consisted of docetaxel 75 mg m^−2^ (day 8) and gemcitabine 1000 mg m^−2^ (days 1+8), every 21 days for a total of six to nine cycles. A total of 31 patients with urothelial bladder cancer, 25 men and six women, aged 42–74 (median 64) years were enrolled. The majority of patients had a good PS (51.6%; PS 0). In all, 15 (48.3%) patients had locally advanced or recurrent disease only and 16 (54.8%) presented with distant metastatic spread, with multiple site involvement in 22.5%. Toxicity was primarily haematologic, and the most frequent grade 3–4 toxicities were anaemia 11 (6.7%) thrombocytopenia eight (4.9%), and neutropenia 45 (27.6%), with 10 (6.1%) episodes of febrile neutropenia. No toxic deaths occurred. A number of patients had some cardiovascular morbidity (38.7%). Nonhaematological toxicities except alopecia (29 patients) were mild. Overall response rate was 51.6%, including four complete responses (12.9%) and 12 partial responses (38.7%), while a further five patients had disease stabilisation (s.d. 16.1%). The median time to progression was 8 months (95% CI 5.1–9.2 months) and the median overall survival was 15 months (95% CI 11.2–18.5 months), with 1-year survival rate of 60%. In conclusion, this schedule of gemcitabine and docetaxel is very active and well tolerated as a first-line treatment for advanced/relapsing or metastatic urothelial carcinoma. Although its relative efficacy and tolerance as compared to classic MVAC should be assessed in a phase III setting, the favourable toxicity profile of this regimen may offer an interesting alternative, particularly in patients with compromised renal function or cardiovascular disease.

Although advanced urothelial carcinoma is a common and relatively chemosensitive neoplasm, it still remains a fatal disease. Over the last 10 years or so chemotherapy of advanced urothelial tumours has focused on cisplatin-based combinations such as cisplatin–methotrexate–vinblastine (CMV), or methotrexate–vinblastine–adriamycin–cisplatin (M-VAC) (von der Maase, 2002). Response rates with standard cisplatin-based combination chemotherapy range from 40 to 70%; however, approximately 50% of all patients will develop metastasis, and recent studies indicate that the disease-free long-term (5-year) survival rate is only about 4% (Sternberg *et al*, 1989; Loehrer *et al*, 1992; Saxman *et al*, 1997). Standard therapy with M-VAC offers a moderate median survival of 1 year; however, it is achieved at the expense of major toxicities, including myelosuppression, nausea, vomiting and nephrotoxicity that often limit its use to patients with normal renal function and adequate performance status (Vaughn, 1999). A recent phase III study has indicated that the combination of gemcitabine/cisplatin could replace the standard of care M-VAC (von der Maase *et al*, 2000; Hussain *et al*, 2002; de Wit, 2003; Raghavan, 2003) since the efficacy was similar in the two regimens with respect to response, time to progressive disease and overall survival; however, toxicity was significantly less in the gemcitabine/cisplatin arm (von der Maase *et al*, 2000). Owing to the discouraging long-term survival data for M-VAC and gemcitabine/cisplatin, attempts at minimising toxicity and maintaining current median survival times are recognised as important considerations in patient management since palliation appears to be a current goal for urothelial cancer.

Therefore, research is now focusing on new regimens including a series of agents that may improve the efficacy of established therapies, while also being effective in the subgroups of patients in which cisplatin-based regimens are contraindicated. Recent studies have indicated that the taxanes (docetaxel and paclitaxel) have significant antitumour activity as single agents (Roth *et al*, 1994; Dreicer *et al*, 1996; McCaffrey *et al*, 1997; Papamichael *et al*, 1997), or when administered in combination with other drugs (Redman *et al*, 1998; Zielinski *et al*, 1998; Bajorin *et al*, 2000; Dreicer *et al*, 2000; Hussain *et al*, 2001), in urothelial cancer. The majority of these new agents have demonstrated synergy when used in combination with a platinum salt showing encouraging response rates of up to 50% and median survival times of 9–14.3 months are reported across a range of combinations (Redman *et al*, 1998; Zielinski *et al*, 1998; von der Maase *et al*, 1999; Dreicer *et al*, 2000; Kaufman *et al*, 2000a; Lorusso *et al*, 2000; Small *et al*, 2000).

We have therefore decided to investigate the efficacy and toxicity of the combination of docetaxel and gemcitabine in chemonaive patients with locally advanced or metastatic urothelial carcinoma.

## PATIENTS AND METHODS

### Patient selection

This was a phase II study in patients with histologically confirmed advanced urothelial cancer. Patients with locally advanced or metastatic cancer of the urothelial tract with transitional cell histologies, and with evidence of measurable or evaluable disease were eligible. No previous chemotherapy for recurrent disease was allowed. Previous neoadjuvant or adjuvant treatment was allowed as long as there was at least a 12-month treatment-free interval and gemcitabine was not part of the previous chemotherapy combination (previous treatment with docetaxel as part of adjuvant or neoadjuvant chemotherapy was allowed provided ⩾12 months had passed).

Patients with other malignant tumour or tumour history, except for nonmelanoma skin cancer or radically excised *in situ* carcinoma of the uterine cervix were excluded. Patients aged >75 years were excluded, as were patients with severe chronic obstructive lung disease. Also excluded were patients with known CNS metastases, patients who were pregnant or those with creatinine clearance less than 50 ml min^−1^, WBC <3.5 × 10^9^ l^−1^, neutrophil count <1.5 × 10^9^ l^−1^ or platelet count <100 × 10^9^ l^−1^ within the 2 weeks preceding the start of the study. Furthermore, patients with active infections or other serious underlying medical or mental conditions, which would impair their ability to receive protocol treatment, could not participate.

The study was conducted according to the Declaration of Helsinki and the guidelines for Good Clinical Practice. The local ethics committees approved the protocol and informed consent was obtained from all patients prior to study entry.

### Treatment schedule

Treatment was administered on an outpatient basis. Gemcitabine 1000 mg m^−2^ was administered by an intravenous infusion over 30 min on days 1 and 8, while docetaxel (75 mg m^−2^) was administered as an intravenous infusion over 1 h on day 8. Treatment was repeated every 21 days. Standard antiemetic premedication and treatment was administered. Haematopoietic growth factors were not used prophylactically. Supportive care, including blood transfusions, analgesics and antiemetics, was administered as appropriate. Patients received a total of six cycles unless disease progression or unacceptable toxicity occurred. Patients who showed disease stabilisation or response were scheduled to receive the same regimen for up to nine full chemotherapy cycles.

### Dose modifications for adverse events

Toxicity was evaluated before each treatment cycle according to the National Cancer Institute Common Toxicity Criteria (NCI CTC version 2.0). Dosage adjustments were made before each treatment based on blood counts, renal and liver function tests and other toxicity. Once the dose of chemotherapy was reduced, they were not re-escalated. Both drugs were given on schedule providing that ANC was ⩾1.5 × 10^9^ l^−1^ and the platelet count ⩾100 × 10^9^− l^−1^. For ANC of <1.5 × 10^9^ l^−1^ on day 1, treatment was delayed for 1 week, haematopoietic growth factor (G-CSF) × 5 days were given, and the next cycle began on day 28 instead of day 21. If platelet counts were <75 × 10^9^ l^−1^ for 1 week, the next cycles were to be given every 4 weeks. If platelets were <75 × 10^9^ l^−1^ for 2 weeks, then the next cycles were to be given every 4 weeks with the doses of both drugs reduced by 25%. If ANC did not recover above 1.5 × 10^9^ l^−1^ for 3 weeks or if platelets were <75 × 10^9^ l^−1^ for >2 weeks, treatment was discontinued and the patient was taken off study. In case of haematological toxicity on day 8 (ANC<1.5 × 10^9^ l^−1^, PLT<100 × 10^9^ l^−1^), the gemcitabine treatment of day 8 was delayed for 1 week. The same dose was then administered on day 15, and the cycle was repeated every 28 days instead of 21 days. In case of persistent haematological toxicity on day 15, the gemcitabine day 8 treatment was omitted and chemotherapy was repeated on day 21 with a 25% dose reduction for both drugs. In case of grade 3–4 neutropenia during a chemotherapy cycle, G-CSF × 5 days was to be used prophylactically for the next cycles, days 10–14.

### Evaluation of response

Baseline evaluation prior to chemotherapy initiation included a complete past medical history, thorough physical examination, a chest X-ray followed by CT scan of the chest if CXR was abnormal, a CT scan of the abdomen and pelvis, bone scan, as well as complete blood counts, renal and liver function tests. During treatment, renal and liver function tests were carried out before each cycle on day 1, and complete blood count was carried out on days 1 and 8 of each cycle. Complete blood counts were also obtained on day 14 of the first course in order to assess nadir WBC and PLT.

Restaging and tumour measurements were performed after three cycles and following completion of treatment, that is, after six or nine cycles of chemotherapy, unless earlier evaluation was required. Dose intensity was defined as the total amount of the drug given (mg m^−2^) divided by the number of weeks. All patients were analysed on an intention-to-treat basis. Therefore, all patients, even those receiving only one cycle of chemotherapy, were analysed for toxicity, response, duration of response and survival. Patients who discontinued treatment before an evaluation was performed were rated as nonresponders. Standard WHO criteria were used to assess response.

Treatment was discontinued if disease progression was documented, or when unacceptable toxicity or conditions requiring therapeutic intervention not permitted by the protocol occurred. Also, treatment was discontinued when the patient wished to withdraw consent, or in any other situation where, in the opinion of the investigator continued participation in the study would not be in the best interest of the patient. All adverse events resulting in discontinuation of study drug were followed closely until resolution or stabilisation. Follow-up disease evaluation was performed regularly at 3 monthly intervals after treatment completion till death. Progression or disease relapse, surgical procedures (cystectomy, etc.), site of relapse and cause of death were noted.

### Statistical analysis

The study was a nonrandomised, phase II study. The primary end point was objective response rate and secondary end points were overall survival, TTP and toxicity. The sample size was calculated on the assumption that a 40% response rate would be detected and the minimum acceptable response rate would be 20%. According to Simon's two-stage design, a sample of 18 patients was required in the first step. If a minimum of five responses were observed, a total of 33 patients would be accrued. Thereby, if at least 11 responses occurred, the probability of accepting a treatment with a real response rate of less than 20% would be 5%. On the other hand, the risk of rejecting a treatment (at the second stage) with a response rate of more than 40% would be 20%. Time to disease progression (TTP) was calculated from the initiation of treatment to the date progression of the disease was firstly documented (patients who discontinued their treatment for any reason or died from probable disease – related causes were considered, at that time, as having disease progression). Survival was calculated from initiation of treatment to the date of last contact or to the date of death. The Kaplan–Meier method (Kaplan and Meier, 1958) was used to calculate TTP and survival curves and exact CIs (Lentner, 1982) were used to determine the 95% upper and lower confidence limits of response rate. Data analysis was performed using SPSS 10.5 (SPSS, Inc, Chicago, IL, USA).

## RESULTS

### Patient characteristics

Between May 2000 and April 2002, 31 patients with locally advanced/relapsed or metastatic urothelial carcinoma of stage IV were accrued to this single-institution study. Two patients could not be evaluated for efficacy end points since they were lost to follow-up after two and three cycles, respectively. Final data analysis was performed in May 2003, 12 months after accrual of the last patient. The main patient baseline characteristics are summarised in Table 1. The majority of patients had ECOG Performance Status of 0 or 1; only 9.7% of the patients had a PS of 2. A percentage of the patients (12; 38.7%) had some type of coexisting cardiovascular disease.

More than half of the patients presented with distant metastases, while among the remaining 15 patients, five (16.1%) had locally recurrent and 10 (32.2%) had *de novo* locally advanced disease. The total number of patients with disease in the pelvis was 20 (64.5%). Eight (25.8%) patients presented both local and distant disease, with multiple site involvement in seven (22.5%) patients. Study treatment was administered in all cases as first-line therapy; however, while 14 patients had not previously received any therapeutic manipulation, 16 patients had prior surgery, eight patients were exposed to prior adjuvant/neoadjuvant chemotherapy, or radiotherapy (*n*=8) to the pelvis. Eight patients had previously received chemotherapy in the adjuvant or neoadjuvant setting; two with M-VAC, one with MVEC, two with CMV, one with CisCA and two with carboplatin/gemcitabine.

### Study treatment administration

All patients were included in the analysis of treatment administration and toxicity. A total of 163 treatment cycles were administered. Patients received a median number of six cycles of chemotherapy, with a range of one to nine cycles. Of the 31 patients analysed, 20 patients (64.5%) completed at least six cycles of treatment, and six (19.4%) received seven to nine cycles. Reasons for treatment discontinuation included disease progression or recurrence in eight patients. Dosage of study drugs was modified mainly due to haematological toxicity. A total of 20 patients received G-CSF. Gemcitabine doses were reduced (dose actually taken ⩽90% of planned dose) or omitted on day 1 in 15 (9.2%) cycles and on day 8 in nine (5.5%) courses. The median delivered dose intensity was 599.4 mg m^−2^ week^−1^ (range: 580–633 mg m^−2^) for gemcitabine, and 20.2 mg m^−2^ week^−1^ for docetaxel (range: 15–25 mg m^−2^), with a mean relative dose intensity of 90 and 95% for gemcitabine and docetaxel, respectively. All five patients with bone metastasis received pamidronate 90 mg every 3–4 weeks for 6–12 months to achieve palliation of skeletal symptoms.

### Toxicity

All patients (*n*=31) were evaluable for toxicity. Toxicity was primarily haematologic with neutropenia being the most prominent with 33 incidences of grade 3 and 12 of grade 4. There were 10 episodes of grade 4 febrile neutropenia requiring hospitalisation. No toxic deaths occurred. Nonhaematological toxicities were mild, 23 episodes of hypersensitivity and/or cutaneous reactions grade 3/4. Alopecia occurred in nearly all patients. Grade 3–4 toxicity data according to the NCI CTC criteria are summarised in Table 2.

### Response and survival

From a total of 31 patients entered in the study, two were lost to follow-up, however, due to the intention-to-treat-analysis; all of the patients enrolled were included in the main analysis of the response rate; Table 3. Four patients achieved a complete response (CR 12.9%) and 12 patients achieved a partial response (PR 38.7%), giving an overall response (OR) of 51.6%. In addition, five patients achieved disease stabilisation (s.d. 16.1%), while eight patients had progressive disease (PD 25.8%). Responses were seen at all sites of measurable disease. Skeletal sites were not included in the response assessment given the concurrent use of pamidronate, although in all five patients with bone metastases a symptomatic palliation as well as evidence of stabilization (three patients) or bone healing (two patients) were noted.

**Figure 1:**
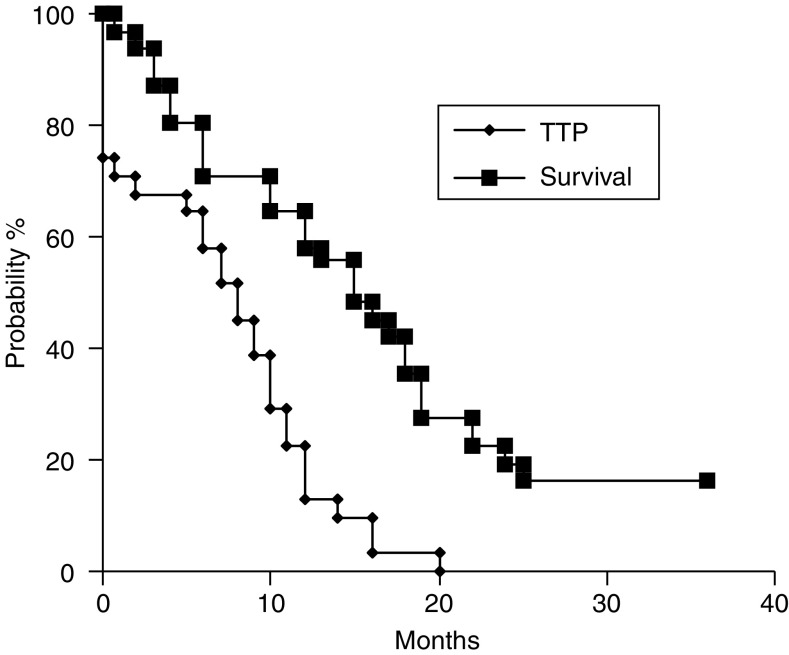
Time to progression (TTP) and overall survival.

With a median follow-up of 23 months (range 12–36 months), 26 patients have died (83.9%). The median time to progression (TTP) was 8.0 months (range 0.0–20.0, 95% CI: 5.1–9.2). With five patients still alive, two at 36+ months the median survival has not been reached; 15 months (range 2.0–36.0+, 95% CI: 11.2–18.5). The estimated 1-year survival rate was 60% (Figure 1).

## DISCUSSION

In this phase II trial, the combination of gemcitabine and docetaxel was well tolerated and effective as first-line treatment for metastatic urothelial carcinoma. The OR rate of 51.6% and a median overall survival of 15 months were similar to a series of rates achieved with combinations including cisplatin/gemcitabine (Moore *et al*, 1999; von der Maase *et al*, 1999; Kaufman *et al*, 2000a; Lorusso *et al*, 2000) or cisplatin/docetaxel (Sengelov *et al*, 1998; Dimopoulos *et al*, 1999).

For many years the gold standard treatment for urothelial cancer has been the M-VAC regimen. However, with the results of the recent randomised comparison with gemcitabine/cisplatin, options are rapidly changing (von der Maase, 2002). It has been realised that using alternative two or three drug combination regimens principally utilising platinum salts, efficacy with respect to disease-free survival, time to progression and overall survival can be maintained while reducing not only the toxicity but also improving the overall quality of life (Sternberg, 2000). There are, however, concerns with the use of platinum salts, as the toxicities are not insignificant especially considering that long-term survival remains relatively poor and the major clinical benefit for the move away from M-VAC is palliative rather than curative. As many patients are unable to tolerate cisplatin-based therapy, carboplatin has been investigated as an alternative. Although it has found its way into clinical use due to a low toxicity profile, when compared to standard M-VAC, trials indicate disappointing single-agent response rates (Medical Research Council, 1987) and shorter median survival when combined with paclitaxel (Redman *et al*, 1998; Vaughn *et al*, 1998). In the few studies that have been published, the combination of carboplatin/gemcitabine appears to be inferior to that obtained with gemcitabine/cisplatin (Stadler, 2002; von der Maase, 2002).

Although few studies have investigated the combination of gemcitabine/paclitaxel, the so far reported small phase II trials have indicated that the combination is effective and well tolerated. In a phase II study using 2500–3000 mg m^−2^ gemcitabine and 150 mg m^−2^ paclitaxel every 2 weeks in M-VAC-pretreated patients, Marini *et al* (1999) reported an overall response rate of 62% and complete response of 24%. Neutropenia grade 3/4 was noted in 43% and neurotoxicity in 6% of the patients. In the studies by Sternberg *et al* (2000) and Kaufman *et al* (2000b), combination of 3000 mg m^−2^ gemcitabine and 150 mg m^−2^ paclitaxel was delivered every 2 weeks in previously treated and chemonaive patients, respectively. They reported OR rates of 39 and 64% (CR 25%), respectively. In a further study by Meluch *et al* (2001), gemcitabine was administered on days 1, 8 and 15 at 1000 mg m^−2^ and paclitaxel at 200 mg m^−2^ on day 1 every 21 days. In all, 29 of 54 eligible patients responded (54%), 22 in the group of 42 patients with no prior chemotherapy, with a CR rate of 7%. Grade 3/4 neutropenia was observed in 46% and grade 3/4 thrombocytopenia was seen in 13% of the patients.

As for the combination of gemcitabine/docetaxel in urothelial cancer, only one report has been published in which three out of five patients developed pulmonary toxicity in a phase I setting (Dunsford *et al*, 1999). In our patients no clinically significant toxicity was detected, although there might have been some subclinical decrease of respiratory reserve. However, none of our patients had a history of lung disease except for smoking, continued or in the past, in 19 patients. Nevertheless, the risk of pulmonary toxicity must be kept in mind when treating patients with this combination. The absence of cardiovascular complications in this population, although not expected with the gemcitabine/docetaxel combination, has to be emphasised, since a significant proportion of patients presented with some heart or arterial disease.

To our knowledge, the present clinical trial represents one of the first phase II studies using this combination showing an OR of 51.6% and CR of 12.9% comparable with other studies in chemotherapy naïve patients, albeit mainly using the combination of gemcitabine/paclitaxel. Moreover, one recent phase II study has investigated the combination of gemcitabine/docetaxel in TCC with a different schedule and found a lower objective response rate (33.3%) but a similar overall (52 weeks) survival (Gitliz *et al*, 2003).

The taxanes are primarily being studied due to their efficacy in previously pretreated patients. Incorporation of taxanes as first-line treatment for advanced urothelíal carcinoma may not only offer an alternative to gemcitabine/cisplatin but may eventually be indicated as a first-line treatment, if equivalence to M-VAC or gemcitabine/cisplatin regimens can be confirmed by further studies. At present the choice of effective regimens needs to be addressed by more rigorous patient stratification and comparison with M-VAC or gemcitabine/cisplatin in both naïve and previously pretreated patients, while special attention should be paid towards compromised patients.

In conclusion, the efficacy of the gemcitabine/docetaxel regimen reported in this study coupled with the acceptable toxicity observed indicate that this combination is an interesting candidate for future comparisons with M-VAC or other gemcitabine- or cisplatin-based regimens. Furthermore, the favourable toxicity profile indicates it as an interesting alternative, particularly in patients with compromised renal function or cardiovascular disease.

## Figures and Tables

**Table 1:** Patient characteristics

	**Patients**	
**Characteristic**	**No.**	**%**
Number of patients registered	31	
*Age, years*		
Median	64	
Range	42–74	
		
*Sex*		
Male	25	80.6
Female	6	19.4
		
*Performance Status ECOG*		
0	16	51.6
1	12	38.7
2	3	9.7
		
*Cardiovascular comorbidity*		
Hypertension (controlled)	18	58
Atherosclerosis	11	35.5
Coronary artery disease	7	22.5
Total (one or a combination of the above)	12	38.7
		
*Histological types*		
Transitional cell carcinoma	31	100
		
*Previous treatment*		
Surgery	16	51.6
Chemotherapy	8	25.8
Radiotherapy	8	25.8
		
*Sites of disease*		
Locally advanced disease	10	32.2
Locally recurrent	5	16.1
*Metastatic disease*	16	51.6
Lymph nodes	7	22.5
Bones	5	16.1
Lung	9	29.0
Liver	7	22.5
Multiple site involvement	7	22.5
Local and distant disease	8	25.8

Note: patients can have more than one site of metastatic disease.

**Table 2:** Toxicity (cycles *n*=163)

	**Grade 3**		**Grade 4**	
**Toxicity**	**No.**	**%**	**No.**	**%**
Anaemia	11	6.7	0	0
Neutropenia	33	20.2	12	7.4
Thrombocytopenia	6	3.7	2	1.2
Diarrhoea	10	6.1	0	0
Febrile neutropenia	0	0	10	6.1
				
Cutaneous	11	6.7	3	1.8
Hypersensitivity	9	5.5	0	0
Fever	9	5.5	0	0
Oedema	8	4.9	0	0
Mucositis	7	4.3	1	0.6

Percentages expressed per cycle.

**Table 3:** Response and survival (*n*=31)

	**No.**	**%**
Complete response (CR)	4	12.9
Partial response (PR)	12	38.7
Stable disease (SD)	5	16.1
Progressive disease (PD)	8	25.8
Overall response (OR)	16	51.6
		
	**Median (months)**	**95% CI**
Time to progression	8.0	5.1–9.2
Overall survival	15	11.2–18.5
